# Similar Attitudes, Different Strategies: A Limited Survey of the Discourse Strategies to Oppose Genetically Modified Organisms Conspiracy Theories by Chinese Scientist Communicators and Citizen Communicators on Zhihu

**DOI:** 10.3389/fpsyg.2022.926098

**Published:** 2022-07-22

**Authors:** Zheng Yang

**Affiliations:** Center for Chinese Urbanization Studies, School of Communication, Soochow University, Suzhou, China

**Keywords:** GMOs conspiracy theories, citizen science communicators, scientist science communicators, online science communication, discourse strategies

## Abstract

The development of the digital media environment has led to a diversification in the role of science communicators. Both scientists and non-scientist citizens can act as science communicators in relation to online discussion of genetically modified organisms (GMOs). Through a limited study, based on thematic and open coding of 60 answers provided by scientist science communicators and citizen science communicators on GMOs on Zhihu, the biggest Chinese knowledge sharing network, it has been found that “business conspiracy theories” about GMOs are the most mentioned and discussed theories, followed by the conspiracy theory of “GMOs as state control tool” on Zhihu. Both citizen science communicators and scientist science communicators are inclined to show oppositional attitudes to GMOs conspiracy theories on Zhihu, despite the differences in their scientific backgrounds; however, they use very different discourse strategies. Citizen science communicators tend to use “lay logic” with more rhetoric, while scientist science communicators tend to use direct scientific knowledge and logic with less rhetoric.

## Introduction

From their earliest conception, genetically modified organisms (GMOs) have been widely discussed in public and media reports, giving rise to several conspiracy theories (Burke, 1999; Lyons et al., 2019; Evanega et al., 2022). In China, a country with a more conservative attitude toward crops and food, although its public’s acceptance of GMOs is higher than other GM producers (around 40%, Zhao et al., 2019), such as United States, Argentina, Brazil, India, negative discussions and conspiracy theories about GMOs are still widespread (Chameides et al., 1999; Yang et al., 2014; Li et al., 2019; Wang et al., 2021). These conspiracy theories have affected the promotion of GMOs and formulation of relevant policies in China (Cao, 2018; Jiang and Fang, 2019; Li et al., 2019). Discussions of GMOs conspiracy theories seem to be further diffused in the digital media environment, as the public has more discourse power and the possibility of becoming communicators based on the empowerment afforded by digital media (Jiang and Fang, 2019; Li et al., 2019). Lay citizens are often singled out as the major believers and communicators of such conspiracy theories as they have limited scientific literacy and ability to identify conspiracy theories (Xu and Lu, 2019; Yang et al., 2021). Is this still the case in China’s current network society? To answer this question, this study explores the attitudes toward current popular GMOs conspiracy theories of Chinese scientist science communicators and citizen science communicators. It also explores the discourse strategies used to defend attitudes toward GMOs conspiracy theories on Zhihu, the biggest Chinese knowledge-sharing network, based on an open coding of the answers provided by these two kinds of science communicators in the GMOs section on Zhihu.

## Genetically Modified Organism Conspiracy Theories in China

Conspiracy theories about GMOs have been around since their birth and have already become one of the important factors influencing GMO promotion, making it a controversial topic (Lewandowsky et al., 2013). According to Douglas et al. (2019), “‘Conspiracy theories’ are attempts to explain the ultimate causes of significant social and political events and circumstances with claims of secret plots by two or more powerful actors” (p. 4). Conspiracy theories occur when an event or situation is seen as the result of a secret plan by powerful actors, even though other possible, plausible, explanations exist (Keeley, 2019). GMOs conspiracy theories relate to the production, trade and consumption of GMOs and foods (Uscinski and Parent, 2014).

There are four main types of popular GMOs conspiracy theories: (1) GMOs as biological weapons, whereby genetically modified technology transfers bacterial and viral genes into animal and plant cells, with the resultant GMOs sold to other countries to harm their citizens (Bielecka and Mohammadi, 2014; Zhang, 2016). (2) GMOs as state control tool, which believes GMOs are a tool of Western developed countries, especially the United States, to control developing nations and the world’s food supply. These theorists believe that developing countries are increasingly reliant on genetically modified technology and crops developed by developed Western countries, thus their food strategy and security are tightly controlled by developed countries (Lynas, 2013; Smith, 2016; Li et al., 2019). (3) Business conspiracy theories, which believes that GMOs are a ploy by giant international agrichemical corporations such as Monsanto to sell more pesticides or herbicides. Conspiracists suggest that GMOs use more insecticides, thus giant international agrichemical corporations can make more money from selling pesticides or herbicides by promoting the cultivation of genetically modified crops (Lynas, 2013). (4) Genocide conspiracy theories, which believe that GMOs technology is a bioweapon used by White people to wipe out other people of color – this theory was popularized after an outbreak of the Zika virus, which was suspected of being produced and spread by genetically modified mosquitoes (Smallman, 2018; Mitchell, 2019).

The types of GMO conspiracy theories described above are also widespread in the Chinese context, especially “GMOs as state control tool” and “GMOs as biological weapons.” According to Cui and Shoemaker (2020), more than 45% of Chinese respondents believed that “GMOs is a huge conspiracy, a tool used by Monsanto Corporation and the United States government behind it to destroy Chinese agriculture, and further a biological weapon against developing countries” (p. 155). Some Chinese public figures, like economist Xianping Lang, have linked negative social news in China, such as “more than half of the college students in Guangxi province have infertile semen,” and ‘the southwest of China suffers from severe drought’ to the promotion of GMOs, which has further fuelled the spread of conspiracy theories in China (Liu, 2012). According to Liu’s observation (Liu, 2012), many Chinese believe that GMOs are a biological weapon used by the United States to wage future wars, to control and even destroy other nations and races. Some Chinese scholars think that the prevalence of GMOs conspiracy theories in China is mainly due to the long-term political confrontation between China and the West, especially the United States, the politicization of food and GMOs, Chinese people’s naturalism, nostalgia and old-fashioned mood on food, and the Chinese lack of trust in institutions such as science and government (Fan, 2014; Cui and Shoemaker, 2020; Yang, 2021a).

The rapidly developed digital environment is considered to have fuelled the amplification and dissemination of conspiracy theories, including those that are GMOs related (Mahl et al., 2022), especially since it gives the public a greater voice and the possibility of becoming communicators based on the empowerment of the digital media environment (Hussein et al., 2020; Mahl et al., 2022). When the growth of the public’s scientific literacy cannot keep up with the growth of their voice power in the digital age, Xu and Lu (2019) found that conspiracy theories about GMOs are more likely to appear on the Chinese Internet. Other scholars have found that the professionalism and credibility of the Chinese public (non-scientists) as communicators of GMOs are not inferior to those Chinese scientists who are considered to be an effective and active force against GMOs conspiracy theories (Yang, 2021a,b). As science communicators, the Chinese public has become a powerful force against conspiracy theory and rumors, and further delivers accurate scientific information in the discussion of GMOs in the Chinese digital environment (Yang, 2021a,b).

## Citizen Science Communicators and More Diverse Science Communicators in Online Genetically Modified Organism Communication

In the digital media environment, science communication about GMOs has been found to be diverse, especially in relation to those who act as science communicators (Dickel and Franzen, 2016; Jia et al., 2017; Yang, 2021a,b). In China, the role of science communicators has been found to include more than just a monopoly by scientists as the public – without a professional scientific background – are also effective and recognized science communicators on many scientific topics, such as GMOs, rare diseases, and climate change (Nerlich et al., 2010; Vicari, 2021; Yang, 2021a,b). Public citizens without professional scientific backgrounds who are actively engaging in the science communication process as communicators have been identified as “citizen science communicators” (Yang, 2021a,b). For instance, a real estate agent with an educational background in sociology actively answered 16 questions about GMOs on Zhihu by the end of March 2022 – this user has been identified as a typical citizen science communicator in the Chinese online science communication system (Yang, 2022a).

On the topic of GMOs, citizen science communicators present a series of characteristics that are different from scientist communicators, such as offering a perspective that is more pluralistic than a purely scientific approach, using a more humorous tone, having an equal perspective to the public, and using more trust mechanisms (Yang, 2021a,b). Although scientist communicators have been found to occupy a central position in online GMOs discussion (Xu et al., 2018; Wang and Song, 2020; Yang, 2022b), citizen science communicators have already played a very important role in online GMOs communication and discussion (Yang, 2021a,b). In science communication, the attitude of science communicators is often believed to have a strong impact on the audience’s attitude toward a specific topic, such as GMOs (Nielsen, 2010; Brownell et al., 2013; Castell et al., 2014; Baram-Tsabari and Lewenstein, 2017). If the communicators believe in conspiracy theories on a certain topic, this will have a significant impact on the audiences’ attitude in the process of communication (Laziæ and Žeželj, 2021). The public, without a scientific background and limited scientific literacy, has generally been regarded as the main constituent of conspiracy believers and one of the culprits in the spread of conspiracy theories in society, such as those about GMOs and vaccines, especially in the Chinese context (Lynas, 2015; Fasce and Picó, 2019; Jia and Luo, 2021; Luo and Jia, 2021; Yang et al., 2021). Therefore, when non-scientist citizens become science communicators on the topic of GMOs especially in the digital environment, are their attitudes toward GMOs conspiracy theories trustable? And whether those attitudes will further affect the other public’s attitudes toward GMOs conspiracy theory? Studies have also found that scientist communicators and citizen science communicators adopt significantly different discourse strategies to persuade their audience during the science communication process (Fähnrich et al., 2020; Yang, 2021a,b). Therefore, we can also assume that scientist communicators and citizen science communicators will also use different discourse strategies to prove their attitude toward GMOs conspiracy theories and further persuade their audience, no matter they have the similar or different attitude toward GMOs conspiracy theories. Combining the discussion of the types of GMOs conspiracy theory above, this study proposes the following three research questions:

RQ1. In the Chinese digital media environment, what kinds of GMOs conspiracy theories are most common?
RQ2. What are the attitudes of scientist communicators and citizen science communicators toward GMOs conspiracy theories?
RQ3. What kinds of discourse strategies do scientist communicators and citizen science communicators use to prove their attitude toward GMOs conspiracy theories?

Answering the three research questions could help to clarify the situation of GMOs conspiracy theories in the increasingly diversified and complex Chinese online science communication system, especially in the online discussions dominated by different science communicators.

## Research Object and Methods

### Research Object

This study uses Zhihu^1^ as a case study to explore GMOs conspiracy theories in the Chinese digital media environment. Zhihu, founded in 2010, is now the biggest Chinese knowledge-sharing network, or Q&A platform, with more than 100 million monthly active users until the end of 2021. This research uses Zhihu as the research platform for two main reasons. Firstly, many citizen science communicators have been found on Zhihu, especially in the GMOs section (Vicari, 2021; Yang, 2021a). According to Yang’s research, citizen science communicators provided more than 60% of excellent answers in the GMOs section on Zhihu, while scientists only provided around 27% (Yang, 2022a). Since the present study aims to explore the different communicators’ attitudes toward GMOs conspiracy theories and the discourse strategies, they adopt to defend their attitudes, Zhihu – which involves both notable citizen science communicators and scientist science communicators in GMOs communication and discussion – can provide sufficient recourse and data. Secondly, Zhihu accommodates more than 3,000 scientific sections (topics), 1.5 million science-related questions, and more than 3 million answers, which makes Zhihu one of the most comprehensive and popular science communication digital platforms in China. Furthermore, compared with other social media platforms like Weibo and WeChat that face more stringent online censorship, due to its target users having a higher education level and its special characteristics of knowledge sharing, the severity of online censorship on Zhihu is relatively weaker. Therefore, Zhihu is a more suitable platform for discussing conspiracy theories in science communication.

The GMOs section on Zhihu was established in February 2011 and has become one of the most active science sections with more than 6,000 questions, attracting more than 400,000 followers by the end of 2021. To explore the attitudes toward GMOs conspiracy theories and the discourse strategies adopted to defend them, this study uses 30 answers provided by citizen science communicators and 30 by scientist science communicators, chosen randomly from 1,000 excellent answers in the Zhihu GMOs section. “Excellent answers” (精品回答) are automatically selected by Zhihu in each topic based on the number of likes, comments, and content quality of the answers, combined with comprehensive algorithmic measurements. The selected excellent answers all feature higher quality content, contain more information, and are the most popular and influential answers in each topic section.

In this study, scientists have been identified as those who had at least a master’s degree in a scientific discipline and were engaged in science-related work, which may not be related to GMOs. This approach was taken because, in China, most science students need to spend 3 years completing their master’s degree and most of their study time involves laboratory work, which makes it easier for those science students with a master’s degree to be accepted and recognized as scientists by both others and themselves. When manually confirming the identities of users as scientists or not, the author first observed the users’ homepages and identity information they provided. Zhihu issues blue marks to those users with verified identities (including education background and employment situation) to prove that the identities and identity certificates provided by them are accurate. For those users who do not provide identity information or whose identity information is not authenticated, the author sent enquiries to them through the private message function on Zhihu to determine whether they were scientists. Sixty randomly chosen GMOs answers provided by citizen science communicators and scientist science communicators constitute the analysis samples in this study.

### Research Methods

This study adopts a research method that combines thematic coding and open coding. Thematic coding involves recording or identifying passages of text or images that are linked by a common theme or idea and indexing the text into categories, thereby establishing a “framework of thematic ideas about it” (Gibbs, 2007). In this study, thematic coding was used to answer RQ1 and RQ2. All expressions of conspiracy theory in the 60 GMOs answer samples were coded as “GMOs as biological weapons,” “GMOs as state control tool,” “business conspiracy theories,” “genocide conspiracy theories,” and others, and answer providers’ attitudes toward those GMOs conspiracy theories were coded as “support,” “oppose,” and “neutral” (as Table 1). Among them, “GMOs as biological weapons” and ‘Genocide conspiracy theories’ need to be further distinguished which both of them treated GMOs as a kind of biological attack tools. In “Genocide conspiracy theories” expressions, there are clear racist tendencies, which the target of the attack or even exterminate was limited to ethnic groups, such as people of color. Under this kind of conspiracy theory, the purpose of GMOs is considered as a premeditated racial destruction operated by White people. But the subject and object mentioned in the conspiracy theory of ‘GMOs as biological weapons’ are relatively flexible. The target of attack can be human or other creatures. And in some GMOs as biological weapons’ cases, the use of such weapons is not premeditated or deliberate but comes from the characteristics of (immature) transgenic technology itself. Therefore, there is a clear distinction between these two kinds of GMOs conspiracy theories, even they both treat GMOs as bioweapons.

**TABLE 1:** Thematic coding books.

	Theme	Description
GMOs conspiracy theories categories	GMOs as biological weapons	Genetically modified technology can transfer bacterial and viral genes into animal and plant cells; these genetically modified crops or foods are sold to other countries as biological weapons against their citizens
	GMOs as state control tool	GMOs are a tool of Western developed countries, especially the United States, to control developing nations and the world’s food supply
	Business conspiracy theories	GMOs are a ploy by some giant international agrichemical corporations, such as Monsanto to sell more pesticides or herbicides
	Genocide conspiracy theories	GMOs technology is a bioweapon used by the White race to wipe out other people of color
	Others	Other GMOs conspiracy theories not mentioned above
Attitude toward GMOs conspiracy theories	Support	User supports mentioned conspiracy theory
	Oppose	User opposes mentioned conspiracy theory
	Neutral	User is neutral about mentioned conspiracy theory

Open coding aims to develop substantial codes based on labeling concepts and defining and developing categories based on their properties and dimensions. Although some scholars have proposed how to deal with GMOs conspiracy theories, such as improving public media literacy and their scientific literacy, using more facts and science-focused corrections, organizing public participation activities, and so on (Lynas, 2013; Douglas et al., 2019), few studies clearly indicate the discourse strategies people use to support or oppose such theories. There are no ready-made coding guidelines that can be directly used in this study for reference. Therefore, this study adopts the method of open coding for RQ3, retaining the flexibility of coding, mainly referring to the trust generation mechanism: based on reputation; based on mechanism and system; based on social similarity, etc. (Zucker, 1986). The mechanism of trust generation, or the discourse strategies that can be used to generate trust, is like the discourse strategies used to protect someone’s particular attitude or point of view. Therefore, this study takes this as the main reference basis, but still maintain the flexibility of public coding.

### Findings

#### More Discussion Around Business Conspiracy Theories

Through the thematic coding of 60 GMOs answers provided by citizen science communicators and scientist science communicators on Zhihu, it can be found that GMOs conspiracy theories have gained some traction in GMOs discussions on Zhihu. In citizen science communicators’ and scientist science communicators’ answers, conspiracy theories were mentioned 16 and 11 times, respectively (some answers contained more than one kind of theory, which were coded based on the number of conspiracy theories) (Table 2). Obviously, it can be found that more answers provided by scientist science communicators and citizen science communicators do not involve conspiracy theories, that is to say, more scientist science communicators and citizen science communicators are not inclined to discuss relevant contents of GMO conspiracy theories, regardless of whether they support those conspiracy theories or opposes them. Among those answers involving conspiracy theories, citizen science communicators are somewhat more concerned with GMOs conspiracy theories than scientist science communicators on Zhihu. Some studies have also shown that conspiracy theories are more likely to spread among the public rather than among scientists (Gough et al., 2014; Lakhvich, 2021).

**TABLE 2:** Frequency of discussion about different GMOs conspiracy theories on Zhihu.

	Citizen science	Scientist science	Total
	Communicators	Communicators	
GMOs as biological weapons	4 (14.8%)	2 (7.4%)	6 (22.2%)
GMOs as state control tool	5 (18.5%)	4 (14.8%)	9 (33.3%)
Business conspiracy theories	7 (25.9%)	5 (18.5%)	12 (44.4%)
Genocide conspiracy theories	0 (0%)	0 (0%)	0 (0%)
Other	0 (0%)	0 (0%)	0 (0%)
Total	16 (59.3%)	11 (40.7%)	27 (100%)

Among the four defined GMOs conspiracy theories, it is clear that business conspiracy theories received more attention and discussion in both citizen science communicators’ and scientist science communicators’ answers followed by “GMOs as state control tool” and “GMOs as biological weapons” for both groups (Table 1). The “genocide conspiracy theories” were not represented in the sample; thus, it seems that this kind of theory is not popular in online discussions in China. All the conspiracy theories discussed in the samples can be effectively classified into these four defined GMOs conspiracy theories, except the “Genocide conspiracy theories,” which proves that expect the “Genocide conspiracy theories” which may not be applicable to Chinese social and cultural environment about GMOs discussion, the effectiveness of such thematic classification is still reliable.

In statements by China’s official media, discussions about GMOs conspiracy theories are more generally around “GMOs as state control tool,” for example: “there is a saying circulating on the Internet that GMO is a conspiracy of the western countries to calculate and control China” (China Science Daily: 2021-06-01), or “for a long time, there has been a saying about GMO that GMO is a conspiracy of the west, especially US imperialism, to scam China and the Chinese people” (China Science Daily, 2017-01-24). Indeed, it is also significantly discussed on Zhihu, which is just less that “Business conspiracy theories.” In online discussions dominated by individual digital media users, discussions around “Business conspiracy theories” occur more often than discussions around “GMOs as state control tool” and other GMOs conspiracy theories. There seems to be a gap between China’s official discussions and public concerns about GMOs conspiracy theories. In the GMOs discussion on Zhihu, both citizen and scientist users tend to base discussions on GMOs being a kind of commercial technology, which should firstly belong to some commercial companies, such as Monsanto. Therefore, their discussions would be more inclined to focus on “Business conspiracy theories.” For instance:

*GMO is just a technology, which is no different from computer technology. Monsanto just wants to use this technology to do reasonable business* (Citizen science communicator, No. 6).

#### Similar Opposition Toward Genetically Modified Organisms Conspiracy Theories

Although their backgrounds are different, citizen science communicators and scientist science communicators on Zhihu have shown very similar attitudes toward the GMOs conspiracy theories mentioned in their answers. According to the thematic coding of 60 GMOs answers, except for minimal neutrality toward the GMO conspiracy theories mentioned (3/13 citizens’ answers and 1/11 scientists’ answers), both citizen science communicators and scientist science communicators demonstrated a clear rejection of the GMOs conspiracy theories they mentioned (Table 3). For instance:

**TABLE 3:** Attitudes of citizen science communicators and scientist science communicators toward GMOs conspiracy theories on Zhihu.

	Support	Oppose	Neutral	Total
Citizen science communicators	0 (0%)	13 (48.2%)	3 (11.1%)	16 (59.3%)
Scientist science communicators	0 (0%)	10 (37.0%)	1 (3.7%)	11 (40.7%)
Total	0 (0%)	23 (85.2%)	4 (14.8%)	27 (100%)

*Several posts about GMOs circulated on the Internet are full of conspiracy theories. The more famous saying is: GMOs is the biochemical weapon developed by the United States government (or Freemasonry?) against China as a big killer to destroy the Chinese people. I don’t think you will still believe this even if you have seen any documentary about GMOs* (Citizen science communicator, No. 11).

*Imagine if a conspirator really transfers the highly toxic protein harmful to people into rice, the rice is 100% unsafe, who will eat it and buy it?* (Scientist science communicator, No. 4)

Therefore, unlike what many Chinese official media or even some scholars claim – for example: “many people (in China) on the Internet have a conspiracy theory about GMOs, believing that it is the conspiracy of large western enterprises” (People’s Daily Online, 2013-10-14), “in recent years, GMOs conspiracy theory has been widely spread on the Internet in China” (Gao and Qi, 2020) – at least as communicators, both Chinese non-scientists and scientists are opposed to GMOs conspiracy theories. Even those neutral expressions toward some mentioned GMOs conspiracy theories are only simple descriptions with rational attitude, rather than “widespread” as some Chinese official media or scholars claim, for instance:

*Conspiracy theories or something, I don’t pay much attention to, personally. Whether GMOs are harmful to the human body depends on scientific research, not baseless conjecture* (Citizen science communicator, No. 28).

#### Different Discourse Strategies Toward Genetically Modified Organisms Conspiracy Theories

Through the open coding on the discourse strategies used to support attitudes toward GMOs conspiracy theories on Zhihu, referring to the trust generation mechanism (Zucker, 1986), it has been found that citizen science communicators and scientist science communicators adopt different discourse strategies, although they have very similar oppositional attitudes toward GMOs conspiracy theories.

Scientist science communicators prefer to demonstrate their attitude toward GMOs conspiracy theories by resorting to evidence that is endorsed by science, which is more like institution-based trust production proposed by Zucker (1986; Schilke et al., 2017). “Institution-based trust” refers to the trust generated by the guarantee of various professional materials, bureaucratic organizations and professional institutions (Jin, 2018, p. 160). When proving their attitude toward GMOs conspiracy theories, scientist science communicators suggest that it violates professional scientific knowledge or “science” as a social institution (Hartung, 1951). For instance:

*From the retroviral drugs of HIV to the protein structure of Zika virus, even if there are problems, it is up to our scientists to find solutions. Those keyboard warriors who do not understand science and the history of science at all please do not spread those conspiracy theories* (Scientist science communicator, No. 9).

*The scientists engaged in transgenics believe that this breeding method is more reliable than traditional cross-breeding, because the consequences of transplanting a gene are known and controllable. The whole process meets the scientific requirements. Therefore, there is no need to think like conspiracy theory* (Scientist science communicator, No. 29).

*China’s support-GMOs and reverse-GMOs should become a debate between science and conspiracy theory. All conspiracy theories are anti-science in nature* (Scientist science communicator, No. 11).

As in the above examples, when scientist science communicators express their opposition to GMOs conspiracy theories, their expressions are often direct and employ less rhetoric. However, citizen science communicators prefer to use rhetorical devices such as irony to express their opposition to GMOs conspiracy theories. For instance:

*In order to thwart such genetically modified conspiracy, I honestly suggest that the questioner and all the anti-GMOs people immediately give up all food sold in the market (because you don’t know whether there are genetically modified ingredients in it), and go to the virgin forest immediately to find the original species for cultivation. In this way, you can save mankind. Don’t eat any food on the market! Remember!* (Citizen science communicator, No. 12)

In addition to more rhetorical devices, when trying to support their opposition to GMOs conspiracy theories, citizen science communicators resort to a kind of “lay logic” rather than professional scientific knowledge. “Lay logic” means thinking modes that are learned and used in citizens’ daily life without professional training (Williams, 1983). For instance:

*The reason why GMOs conspiracy theories are so popular is that too many people don’t like to use their brains and like to judge the real world through simple imagination and dramatic deduction* (Citizen science communicator, No. 7).

*If GMOs are really toxic, and Americans consume more of these foods than we do, then they should go extinct first* (Citizen science communicator, No. 16).

*If it is really a conspiracy, then this conspiracy chain must spread all over the world, covering almost all scientists, agricultural companies and government departments. Monsanto? No matter how big, it is just a company, okay? God, does such a big interest group or conspiracy chain really exist? I don’t think so* (Citizen science communicator, No. 6).

In the examples above, the citizen science communicators demonstrate the unreliability of GMOs conspiracy theories from the perspective of ordinary people, instead of resorting to more professional scientific knowledge, terminology or logic. The discourse strategy of lay logic is like the social similarity-based trust production described by Zucker (1986). “Similarity-based trust” refers to the trust generated by the similarities in demographic factors, social values, thinking modes, and behavior logic between communicators and audiences (Jin, 2018, p. 160). Using lay logic as a discourse strategy, the citizen science communicators may achieve greater logical and emotional resonance with a citizen audience based on their similar non-professional backgrounds and way of thinking, as professional scientific knowledge or logic is removed from citizen’s daily lives (Yang, 2021a).

Although citizen science communicators and scientist science communicators both oppose GMOs conspiracy theories, they adopt very different discourse strategies to support their attitudes on Zhihu. Scientist science communicators tend to use direct scientific knowledge and logic to support their views with fewer rhetorical devices, while citizen science communicators are more inclined to use “lay logic” with more rhetorical devices. This reflects two completely different trust generation strategies: trust based on institution and trust based on social similarity.

## Discussion and Conclusion

The public acceptance of GMOs is believed to be influenced by multiple factors, such as public perception of GMOs’ adverse effect on the environmental and/or human health (Ishii and Araki, 2016), trust in science or governments (Pechar et al., 2018), public local knowledge and traditional morality (Motta, 2014), price and market circulation of GMOs (Paull, 2019), as well as the conspiracy theories around GMOs (Burke, 1999; Lyons et al., 2019; Evanega et al., 2022). And among those factors, conspiracy theories around GMOs are considered as one of the most important communicational and psychological factors in public discussion and acceptance of GMOs. If you want to understand the acceptance of GMOs in the society, and the public’s psychological perception of GMOs, the studies on conspiracy theories around GMOs cannot be bypassed. Returning to the research questions proposed above, the results of this limited study based on thematic and open coding show that “business conspiracy theories” about GMOs are the most mentioned and discussed GMOs conspiracy theory, followed by “GMOs as state control tool” on Zhihu. Both citizen science communicators and scientist science communicators are inclined to show an oppositional attitude to all GMOs conspiracy theories on Zhihu, despite their different backgrounds. Although they have similar attitudes toward GMOs conspiracy theories, citizen science communicators and scientist science communicators adopt very different discourse strategies to demonstrate their attitude. Specifically, citizen science communicators tend to use “lay logic” with more rhetorical devices, while scientist science communicators tend to use direct scientific knowledge and logic with fewer rhetorical devices.

Many Chinese surveys and academic studies point out that GMOs conspiracy theories are very popular on China’s Internet, especially among the public. For instance, Fan et al. (2013) analyzed the spread of the genetically modified “golden rice incident” on Weibo in the summer of 2012 and found that GMOs conspiracy theories was the main subject in public communication on the issue. Official reports by many Chinese media mentioned above imply that China’s online public, especially those without a scientific background or with low scientific literacy, tend to believe in GMOs conspiracy theories. However, this study shows that, both non-scientific citizens and scientists on Zhihu tend to reject and refute GMOs conspiracy theories when playing the role of communicators. Echoing the existing research, especially those on the GMO conspiracy theories among the Chinese public (Fan et al., 2013; Liu and Huang, 2020), this study could be believed to have a great external reliability, which results maybe can be applied in a broader digital media environment, more than just Zhihu.

During the communication process, the role of audiences is often accompanied with the status of passive acceptance of information, while the role of communicators requires initiative from the people who play such roles when delivering information (Shaw, 2005; Illingworth, 2017). Such active delivering or communicating behaviors requires the intervention of more energy and more careful thinking (Rowan, 1994; Wick, 2000; Rush Hovde and Renguette, 2017), because being a communicator risks criticism by others and similar social pressures (Rowan, 1994). Therefore, when people intervene in the communication process as communicators, they tend to carefully choose those views that are considered more “correct” by society than being just “audiences,” as per the “social skin” in the theory of “Spiral of Silence” (Asch, 1955; Noelle-Neumann, 1993). Although there may still be some conspiracy theories around GMOs in society, the overall trend is that people are more and more aware of the mistakes and inaccuracy of those conspiracy theories (Berman, 2020). Therefore, when they become science communicators, citizens and scientists should consider the pressure of such “social skin” to express the attitude mainly recognized by mainstream society. This may be why the attitude of both citizen science communicators and scientist science communicators toward GMOs conspiracy theories on Zhihu is consistently oppositional. In addition, the scientific literacy of citizens has also been found to be relevant to their attitude toward conspiracy theories (Fasce and Picó, 2019; Miller, 2020; Jia and Luo, 2021; Luo and Jia, 2021; Yang et al., 2021). The public with high scientific literacy tends to oppose conspiracy theories. Due to its characteristics of knowledge sharing, Zhihu’s users are found to have generally higher scientific literacy than other Chinese social media, such as Weibo, WeChat, or Douyin. Studies have also found that users with higher information and scientific literacy are more inclined to spread information on digital media platforms and become communicators (Peng and Chen, 2021; Yang, 2021a). Therefore, we have reason to assume that citizen science communicators on Zhihu have higher information literacy and scientific literacy than ordinary Chinese Internet users, which also contribute to their attitude of rejection toward GMO conspiracy theories like scientist science communicators on Zhihu.

Based on the discussion above, perhaps a binary opposition between scientists and the public to understand GMOs conspiracy theories is incorrect – the idea that scientists oppose GMOs conspiracy theories and educate the public who believe in them is not accurate. This study shows that the communication system of GMOs conspiracy theories, especially in the digital environment, is more complex and diverse. Both scientists and the public can be communicators and advocates of anti-GMOs conspiracy theories. Differences in scientific background do not necessarily change attitudes and solidify their roles in the anti-GMOs conspiracy theories communication process. However, difference in background may change the discourse strategies adopted in anti-GMOs conspiracy theories communication. Citizen science communicators and scientist science communication both tend to adopt discourse strategies that match their social identity, background and experience. Which strategies are more effective in advocating anti-GMOs conspiracy theories? Such questions still need to be determined in more follow-up empirical studies.

An additional observation is that when scientist science communicators and citizen science communicators mention GMOs conspiracy theories, they both tend to think that other citizens will believe these conspiracy theories, but they are soberly opposed to them. This is akin to the “third-person effect” theory by Davison (1983), which suggests that people tend to think that the information in the mass media (especially persuasive or negative information) may have a greater effect on others than on themselves, based on personal biases (Perloff, 1999). This kind of effect has also been found in conspiracy theories communication (Liu and Huang, 2020). In this study, both citizen science communicators and scientist science communicators saw their own views as rational, while others were more easily influenced by GMOs conspiracy theories.

In conclusion, in the face of GMO conspiracy theories, we need to take a more diversified perspective to consider those non-scientist publics and other potential actors. We should not treat their attitudes toward GMOs conspiracy theories and their roles in the communication of anti-GMOs conspiracy theories with a fixed or stereotyped attitude. We must attach more importance to the public’s roles, which may not be inferior to scientists as communicators in anti-GMOs conspiracy theories. Since this study is only based on a qualitative analysis with limited samples, its conclusion may not be absolutely rigorous, which also requires us to continuously expand the analytical sample and research platform beyond Zhihu in the follow-up research.

## Data Availability Statement

The original contributions presented in this study are included in the article/supplementary material, further inquiries can be directed to the corresponding author.

## Ethics Statement

This study was approved by Ethics Administrator Office of Sociological Studies, The University of Sheffield (Approval Number: 023021 on 23 November 2018). Written informed consent from the participants was not required to participate in this study in accordance with the national legislation and the institutional requirements.

## Author Contributions

The author confirms being the sole contributor of this work and has approved it for publication.

## Conflict of Interest

The author declares that the research was conducted in the absence of any commercial or financial relationships that could be construed as a potential conflict of interest.

## Publisher’s Note

All claims expressed in this article are solely those of the authors and do not necessarily represent those of their affiliated organizations, or those of the publisher, the editors and the reviewers. Any product that may be evaluated in this article, or claim that may be made by its manufacturer, is not guaranteed or endorsed by the publisher.

## Footnotes

1
https://www.zhihu.com/
